# The Role of Circadian Rhythms in Muscular and Osseous Physiology and Their Regulation by Nutrition and Exercise

**DOI:** 10.3389/fnins.2017.00063

**Published:** 2017-02-14

**Authors:** Shinya Aoyama, Shigenobu Shibata

**Affiliations:** ^1^Organization for University Research Initiatives, Waseda UniversityTokyo, Japan; ^2^Laboratory of Physiology and Pharmacology, School of Advanced Science and Engineering, Waseda UniversityTokyo, Japan

**Keywords:** circadian rhythm, clock gene, skeletal muscle, bone, chrono-exercise, chrono-nutrition

## Abstract

The mammalian circadian clock regulates the day and night cycles of various physiological functions. The circadian clock system consists of a central clock in the suprachiasmatic nucleus (SCN) of the hypothalamus and peripheral clocks in peripheral tissues. According to the results of circadian transcriptomic studies in several tissues, the majority of rhythmic genes are expressed in a tissue-specific manner and are influenced by tissue-specific circadian rhythms. Here we review the diurnal variations of musculoskeletal functions and discuss the impact of the circadian clock on homeostasis in skeletal muscle and bone. Peripheral clocks are controlled by not only photic stimulation from the central clock in the SCN but also by external cues, such as feeding and exercise. In this review, we discuss the effects of feeding and exercise on the circadian clock and diurnal variation of musculoskeletal functions. We also discuss the therapeutic potential of chrono-nutrition and chrono-exercise on circadian disturbances and the failure of homeostasis in skeletal muscle and bone.

## Introduction

Various physiological functions, including the sleep wake cycle, body temperature, hormone secretion, and locomotor activity, exhibit circadian rhythms. This time-dependent regulation is driven by an internal circadian clock. In mammals, the circadian clock is divided into two parts, the master clock in the suprachiasmatic nucleus (SCN) of the hypothalamus and peripheral clocks in the peripheral tissues, such as the liver, skeletal muscle and so on, as well as brain areas other than the SCN. The master clock in the SCN acts as a time keeper in the whole body; thus, it integrates and entrains the peripheral circadian clocks by regulating neural and endocrine pathways, such as the sympathetic nervous system and glucocorticoid signaling (Schibler et al., 2003; Shibata, 2004). Light is the major entraining factor for the SCN. On the other hand, the peripheral clocks are entrained by not only the light-dependent regulation of the SCN but also scheduled feeding and scheduled exercise in an SCN-independent manner (Tahara and Shibata, 2013). The molecular mechanisms of circadian clock systems in mammals have been investigated since *Clock* (*Circadian locomotor output cycles kaput*) was discovered in 1997 (King et al., 1997). Circadian rhythm is produced by the transcriptional and translational regulation feedback loop of core clock genes, which include *Bmal1* (*Bain and muscle ARNT-like 1*), *Clock, Per1* (*Period1*), *Per2, Cry1* (*cryptochrome1*), and *Cry2*. CLOCK and BMAL1 are transcriptional factors that have a basic helix-loop-helix PAS domain, and the heterodimer of CLOCK and BMAL1 proteins binds to an E-box binding element in the promoter regions of *Per* and *Cry*, and its binding activates the transcription of these genes (Gekakis et al., 1998). After transcription and translation, PER1/2 proteins localize to the cytoplasm and are phosphorylated by CKIε (Casein kinase Iε) (Lowrey et al., 2000). The phosphorylated PER1/2 proteins are not stable and are degraded by ubiquitination systems. CRY1/2 proteins in the cytoplasm are also degraded by ubiquitination systems via FBXL3 (F-box and leucine rich repeat protein 3) (Busino et al., 2007). The localization of CRY1/2 and PER1/2 to the cytoplasm promotes the formation of the PERs/CRYs/CKIε complex. This complex accumulates in the nucleus and suppresses the transcription of *Pers* and *Crys* by CLOCK and BMAL1. In addition, *Rev-erbs* (*nuclear receptor subfamily 1, group D*) and *Rors* (*RAR-related orphan receptor*) genes are the target genes of the complex of BMAL1 and CLOCK (Preitner et al., 2002; Sato et al., 2004). REV-ERBs and RORs act as the repressor and activator of *Bmal1* and *Clock* transcription, respectively, via binding to an ROR-responsive element (Preitner et al., 2002; Sato et al., 2004). Recent circadian transcriptomic studies revealed that clock genes are expressed in several tissues, and that the expression of rhythmic genes in each tissue occurs in a tissue-specific manner (Miller et al., 2007; Zhang et al., 2014), suggesting that peripheral clocks in each tissue generate the biological rhythm of tissue-specific functions.

Clock genes are also expressed in skeletal muscle and bones of the skeleton, and it is thought that the clock genes regulate muscular- or osseous-specific biological functions (Dudek and Meng, 2014; Mayeuf-Louchart et al., 2015; Chatterjee and Ma, 2016; Yang and Meng, 2016). Skeletal muscle and bone have roles in not only the regulation of locomotion and postural support but also the control of nutritional homeostasis, such as maintaining glucose and calcium levels. Feeding and exercise stimulate these tissues and change their functions, including the maintenance of tissue mass and metabolism. Here, we review and discuss mainly two topics as follows: (1) the role of circadian rhythms in the biological functions of muscles and bone, and (2) the entrainment or regulation of the circadian clock or biological rhythm in skeletal muscle and bone by feeding and exercise.

## Circadian rhythm in skeletal muscles

Most mammalian cells express molecular clock genes and have a circadian clock system. Skeletal muscle cells also express molecular clock genes and show a circadian rhythm expression pattern. Using DNA microarray and RNA-seq methods Zhang et al. reported on the circadian transcriptome of 12 mouse organs and found that most rhythmic genes show an organ-specific pattern (Zhang et al., 2014). The circadian transcriptome of adult mouse skeletal muscle has also been identified by Miller et al., McCarthy et al., and Dyar et al. (McCarthy et al., 2007; Miller et al., 2007; Dyar et al., 2015). A small number, 3.4%, of the expressed genes in skeletal muscle show a circadian rhythm expression pattern (Miller et al., 2007). In addition, it has been reported that the number of rhythmic genes in skeletal muscle depends on the muscle fiber type; 684 rhythmic genes were found in the fast-twitch tibialis anterior muscle, while 1359 were identified in the slow-twitch soleus muscle (Dyar et al., 2015). The phase of expression of many rhythmic genes in skeletal muscle occurs at the mid-point of the subjective active phase (McCarthy et al., 2007; Miller et al., 2007). In liver, the phase expression of many rhythmic genes is different from that in skeletal muscle, the large cluster of phase expression occurs at the mid-point of subjective day and subjective night, respectively. One of the reasons for this may be that scheduled exercise can entrain the circadian clocks in skeletal muscles (Wolff and Esser, 2012), i.e., the phase of rhythmic gene expression in skeletal muscle may be regulated by the rhythm of locomotor activity. These results from circadian transcriptomic studies in skeletal muscle suggest circadian regulation of several muscle functions. For example, *Myod1* (*myogenic differentiation 1*), *Ucp3* (*uncoupling protein 3*), *Atrogin1* (*F-box protein 32*), *Myh1* (*myosin heavy chain 1*) are muscle-specific genes that play roles in myogenesis, muscle lipid utilization, protein metabolism and the organization of myofilaments, respectively. They have shown the circadian rhythms of gene expression, some of which have been shown to be directly regulated by clock genes (Andrews et al., 2010; Zhang et al., 2012). On the other hand, since the rhythmic expressions of *Myod1* and *Atrogin1* in *ad libitum feeding* mice are not observed in fasting mice (Shavlakadze et al., 2013), feeding and fasting patterns may be important factors for the rhythmic expression of muscle specific genes.

## The role of the circadian clock in muscle mass, muscle strength, and myofiber type

The roles of molecular circadian clocks in skeletal muscle mass, strength, and myofiber type are demonstrated with the use of molecular clock gene-deficient or mutant mice (Table 1). Kondratov et al. were the first to report on the effect of the molecular clock on muscle phenotype (Kondratov et al., 2006). Whole body *Bmal1* knockout in mice causes sarcopenia, i.e., age-dependent reduction of muscle mass, thus muscle loss is not observed in young *Bmal1* knockout mice (Kondratov et al., 2006). In recent years, the muscular phenotypes of some *Bmal1* knockout mice have been reported, with time-dependent knockout or tissue-specific knockout (Dyar et al., 2014; Schroder et al., 2015; Harfmann et al., 2016; Schiaffino et al., 2016; Yang et al., 2016). Yang et al. reported that body weight loss and early aging were not observed in tamoxifen-inducible *Bmal1* knockout mice under the treatment of tamoxifen after muscle development (>3 months) (Yang et al., 2016). In addition, Dyar et al. showed that reductions in muscle weight and strength were not observed in inducible muscle-specific *Bmal1* knockout mice once *Bmal1* knockout was induced after development (Dyar et al., 2014). These reports suggest that *Bmal1* expression during development is important for the maintenance of weight gain and muscular strength. In addition to the timing of *Bmal1* expression, the role of intrinsic muscular *Bmal1* in the muscle weight and strength has also been reported (Dyar et al., 2014; Schroder et al., 2015). Muscle-specific *Bmal1* knockout mice showed an increase in muscle weight and a decrease in muscle strength, although the whole body *Bmal1* knockout mice exhibited muscle loss. These reports indicate that while a circadian clock during development regulates muscle mass, an intrinsic skeletal muscular clock may not directly regulate it. Lack of muscle use and the lowering of daily locomotor activity levels reduce muscle mass (Powers et al., 2005). Disruption of *Bmal1* in the whole body, and not in only skeletal muscle, induces the disappearance of an activity rhythm and a reduction in daily locomotor activity levels (Kondratov et al., 2006). In another report, muscle-specific over expression of *Bmal1* partially restores the *Bmal1* knockout-induced reduction of activity levels and body weight loss without improving arrhythmic behavior (McDearmon et al., 2006). In addition, the locomotor activity of muscle-specific *Bmal1* knockout mice, which show an increase in muscle mass, have a normal circadian rhythm, and is increased activity during the active phase (Dyar et al., 2014). These reports suggest that circadian clock-regulated activity levels may have an important role in the growth of skeletal muscle. Taken together, the results suggests that early aging and muscle weight loss in whole body *Bmal1* knockout mice are controlled in non-myofiber cells by *Bmal1* during development. Although the role of *Bmal1* for the developing fetus has not been described, it is possible that the rhythmic expression-independent functions of *Bmal1* in skeletal muscle growth are observed because *Bmal1*, just as other core clock genes, does not show rhythmic expression in the embryo (Dolatshad et al., 2010). Indeed, Lipton et al. found that the phosphorylation of BMAL1 by S6K1 (ribosomal S6 protein kinase 1) regulates translation, independently of its regulatory role in transcription (Lipton et al., 2015), suggesting that the phosphorylation of BMAL1 may affect the synthesis of muscle proteins, which is an important process for muscle growth. Further investigation is required of the role of molecular clocks in skeletal muscle growth.

**Table 1:** **Summary of the muscular and osseous phenotypes in clock gene mutant mice**.

**Genotype**	**Muscular or osseous phenotypes**
*Bmal1* knockout	Muscle
	Age-related muscle loss (sarcopenia) (Kondratov et al., 2006)
	Reduction in muscle fiber size (Kondratov et al., 2006; Chatterjee et al., 2013)
	Fiber-type shift (Dyar et al., 2014; Schroder et al., 2015)
	Disruption of myofiber architecture (Andrews et al., 2010)
	Reduction of mitochondrial volume (Andrews et al., 2010)
	Impaired muscle regeneration (Chatterjee et al., 2015)
	Bone
	High bone mass at young age (Fu et al., 2005)
	Age-related bone loss (Samsa et al., 2016)
	Abnormal bone calcification and arthropathy (McDearmon et al., 2006)
Inducible *Bmal1* knockout mice after development (> 3 months)	Bone
	Normal bone and joint (Yang et al., 2016)
Muscle-specific *Bmal1* knockout	Muscle
	Insulin resistance and glucose intolerance (Dyar et al., 2014; Harfmann et al., 2016)
	Impaired insulin stimulated glucose uptake (Dyar et al., 2014; Harfmann et al., 2016)
	Increased muscle mass and size (Dyar et al., 2014)
	Decreased muscle strength (Dyar et al., 2014)
	Slight shift in fiber type (Dyar et al., 2014)
	Bone
	Thick bone (Schroder et al., 2015)
Muscle-specific inducible *Bmal1* knockout after development (> 3 months)	Muscle
	Normal muscle weight and normal size (Dyar et al., 2014)
	No significant change (Dyar et al., 2014) or a slight decrease in muscle strength (Schroder et al., 2015)
	No significant change (Dyar et al., 2014) or a slight shift in fiber type (Schroder et al., 2015)
Osteoclast-specific *Bmal1* knockout	Bone
	High bone mass (Xu et al., 2016)
*Clock* mutant	Muscle
	The disruption of myofiber architecture (Andrews et al., 2010)
	Reduction in muscle strength (Andrews et al., 2010)
	Reduction in mitochondria (Andrews et al., 2010)
*Per1* knockout	Muscle
	No significant change in muscle mass (Bae et al., 2006)
	Bone
	No significant change in bone mass (Fu et al., 2005)
*Per2* knockout or *Per2* mutant	Muscle
	No change in muscle mass and lower exercise tolerance (Bae et al., 2006)
	Bone
	High bone mass at 3 months of age (Maronde et al., 2010)
	No significant change in bone mass (Fu et al., 2005)
*Per1/2* knockout or *Per1/2* mutant mice	Bone
	High bone mass (Fu et al., 2005)
*Per1^−/−^*/Osteoblast-specific-*Per2* mutant mice	Bone
	High bone mass (Fu et al., 2005)
*Cry1* knockout	Bone
	No significant change in bone mass (Fu et al., 2005)
*Cry2* knockout	Bone
	High bone mass at 3 months of age (Maronde et al., 2010)
	No significant change in bone mass (Fu et al., 2005)
	Bone
	High bone mass (Fu et al., 2005)
*Per2* mutant / *Cry2* knockout	Bone
	No significant change in bone mass (Maronde et al., 2010)
*Rev-erba*α knockout	Muscle
	Disruption of myofiber architecture (Woldt et al., 2013)
	Reduction of mitochondrial volume (Woldt et al., 2013)
	Lower exercise capacity (Woldt et al., 2013)
	Slight fiber-type shift (Pircher et al., 2005)

*Bmal1* knockout mice and *Clock* mutant mice have disrupted myofilament architecture in their skeletal muscle and decreased muscle strength at a single-fiber level (Andrews et al., 2010). The disruption of the myofilament architecture is observed in *Myod* knockout mice as well as *Bmal1* knockout mice (Andrews et al., 2010). There is a binding site for the heterodimer of CLOCK and BMAL1 in the promoter region of *Myod1* and this heterodimer directly regulates the rhythmic expression of *Myod1*, which is not seen in *Bmal1* knockout mice or *Clock* mutant mice (Andrews et al., 2010; Zhang et al., 2012). MyoD plays a role in myogenesis, which includes the formation of myofibers from satellite cells and myoblast cells. Cahtterjee et al. demonstrated that BMAL1 is one of the key players of myogenesis (Chatterjee et al., 2013). Deficiency of *Bmal1* in myoblast cells suppresses myogenesis related-gene expression, including *Myod, Myf5* (*myogenic factor 5*), and *Myogenin* expression, and impairs the differentiation of myoblasts to myofibers (Chatterjee et al., 2013). Moreover, they have shown a role for the Wnt (wingless-type MMTV integration site family) pathway in *Bmal1*-induced myoblast differentiation, since the circadian regulation of Wnt pathway-related genes is regulated by *Bmal1* (Chatterjee et al., 2013). In addition, they have demonstrated that *Bmal1* in skeletal muscle also promotes skeletal muscle regeneration via satellite cell proliferation using *in vivo* muscle injury models (Chatterjee et al., 2015). However, Schiaffino et al. reported that *Myod1* expression in the skeletal muscle of muscle-specific *Bmal1* knockout mice showed rhythmicity and was increased during the active phase (Schiaffino et al., 2016), while a previous study showed that whole body *Clock*^Δ19^ mice did not show rhythmicity (Andrews et al., 2010). As one of the reasons, the up regulation of *Myod1* in muscle-specific *Bmal1* knockout mice may be due to the increase in locomotor activity during the active phase (Dyar et al., 2014); this hypothesis is supported by the finding that mechanical stimulation, such as during exercise, increases *Myod* expression (Legerlotz and Smith, 2008). Indeed, a stimulation such as fasting, which might cause hypolocomotion, down regulated the rhythmic expression of *Myod1* under conditions when the rhythmic expression of *Bmal1* is maintained (Shavlakadze et al., 2013). As another reason, the rhythmic expression of *Myod1* in muscle-specific *Bmal1* knockout mice may be because of the tissue-specific promoter driving genetic ablation. *Myosin light chain 1f* (*Mlc1f*) promoter was used to drive the ablation of muscle-specific *Bmal1* (Dyar et al., 2014). *Mlc1f* promoter is active in mature myocytes, but not in muscle precursor cells, such as satellite cells (Lee et al., 2012), suggesting that deficiency of *Bmal1* occurs only in mature myocytes. *Myod* is expressed not only in muscle precursor cells, such as activated satellite cells and myoblasts, but also in the mature muscle myocytes, albeit at low levels (Voytik et al., 1993; Hughes et al., 1997). The transcriptional regulation of *Myod* occurs through two elements, the “core enhancer” and a bipartite element containing the “distal regulatory region” and the “proximal regulatory region” (Charge et al., 2008). *Myod* expression in myoblasts is activated through the core enhancer, and its expression in mature myocytes is up regulated through the bipartite element (Charge et al., 2008). In addition, BMAL1 and CLOCK bind to the core enhancer, but not the bipartite element (Andrews et al., 2010). The rhythmic expression of *Myod1* in muscle-specific *Bmal1* knockout mice may reflect its rhythmic expression in activated satellite cells and myoblasts, which are included in adult skeletal muscles. Therefore, these reports indicate that clocks in myoblasts and satellite cells and locomotor activity rhythms regulate skeletal muscle functions, including the formation and growth of skeletal muscle, via skeletal muscle specific regulators such as MyoD.

Skeletal muscle fiber is divided into the two types, slow-twitch fiber and fast-twitch fiber, according to the myosin heavy chain isoform composition (Schiaffino and Reggiani, 1996). The slow-twitch fiber is mainly composed of myosin heavy chain isoforms I and IIa. It has high oxidative capacity and mitochondrial volume. On the other hand, the fast-twitch fiber is mainly composed of myosin heavy chain isoforms IIx and IIb. Some circadian clock genes regulate fiber type composition in skeletal muscle. Muscle-specific *Bmal1* knockout mice show a shift in their fiber type from slow- to fast-twitch compared with wild type mice (Dyar et al., 2014; Schroder et al., 2015). Deficiency of *Rev-erb*α, a repressor of *Bmal1* transcription, induces a shift in fiber type from fast- to slow-twitch in the slow-twitch soleus muscle, however, its effects are small (Pircher et al., 2005). On the other hand, Woldt et al. showed that fiber type did not shift significantly in the wild type and *Rev-erb*α mice, although the genetic expression of slow-twitch fiber markers was lower in muscle from *Rev-erb*α mice than in wild type mice (Woldt et al., 2013). The reason for this discrepancy may be in the independent line of the global genetic targeting of the *Rev-erb*α allele. While there are few reports regarding the relationship between molecular clocks and muscle fiber types, *Bmal1* has the potential to shift fiber types from fast- to slow-twitch. In contrast, *Rev-erb*α, the repressor of *Bmal1*, has opposing effects on fiber types.

## The role of the circadian clock on lipid and carbohydrate metabolism in skeletal muscle

As mentioned previously, there is a lot of evidence regarding the regulatory effects of circadian rhythm on lipid and carbohydrate metabolism in the liver (Tahara and Shibata, 2016). In skeletal muscle, circadian rhythm-regulated lipid and carbohydrate metabolism may be due to intrinsic molecular clocks or could be a response to a behavior such as feeding/fasting or neural and hormonal cues. Hodge et al. have reported that *Bmal1* in skeletal muscle-regulated genes is involved in the utilization and storage of energy substrates, independent of circadian activity (Hodge et al., 2015). For example, the peak time of lipogenic and lipolytic gene expression occurs at the end of the active phase and at the middle of the inactive phase, respectively. Carbohydrate catabolism and storage peak at the beginning of the active phase and in the middle of the active phase, respectively. These substrate-dependent peak times for metabolic genes in skeletal muscle are partially controlled by an intrinsic skeletal muscle molecular clock (Hodge et al., 2015). Other reports have demonstrated that insulin-stimulated glucose uptake, its metabolism, and its related factors are down-regulated in skeletal muscle-specific *Bmal1* knockout mice (Dyar et al., 2014; Harfmann et al., 2016). Glucose transporter 4 (GLUT4) is a key molecule for glucose transport in skeletal muscle. Insulin translocates GLUT4 from the cytoplasm to the plasma membrane via the activation of the insulin signaling pathway, which includes TBC1 domain family, member 1 (TBC1D1). Quantities of GLUT4 and TBC1D1 show a diurnal change, with increased levels during the active phase and decreased levels during the inactive phase (Dyar et al., 2014). These diurnal changes are not observed in muscle-specific *Bmal1* knockout mice (Dyar et al., 2014) and a deficiency in *Bmal1* reduces levels of these molecules throughout the day (Dyar et al., 2014; Harfmann et al., 2016). On the other hand, the deficiency of muscular *Bmal1* did not affect insulin signaling, including the phosphorylation of Akt (Dyar et al., 2014). In addition to affecting the transport of glucose, a deficiency in *Bmal1* causes dysregulation of glycolysis and glucose oxidation via the inactivity of metabolic enzymes, such as Hexokinase 2 (HK2) and Pyruvate dehydrogenase (PDH), suggesting abnormal glucose metabolism (Dyar et al., 2014; Harfmann et al., 2016). Owing to the dysregulation of the glycolytic pathway, a deficiency of *Bmal1* in skeletal muscle increases levels of the metabolites related to the pentose phosphate pathway, the polyol pathway, and glucuronic acid pathway (Dyar et al., 2014). These results, which relate not only to the transcriptome, but also to protein levels and metabolite levels, strongly support the notion that a major physiological role of the muscle clock is to prepare for the transition from the rest/fasting phase to the active/feeding phase, when glucose becomes the predominant fuel for skeletal muscle. Another investigation, using C2C12 myotubes, showed the role of muscle clocks in insulin sensitivity through Sirtuin 1 (*Sirt1*) (Liu et al., 2016). In this report, the knockdown of *Clock* and *Bmal1* caused insulin resistance via *Sirt1* (Liu et al., 2016). The muscular clock-regulated *Rev-erb*α also shows high expression levels in slow-twitch fiber type muscle, such as soleus muscle, and regulates lipid uptake and oxidative capacity in skeletal muscle by controlling mitochondrial biogenesis and autophagy (Woldt et al., 2013). A deficiency in *Rev-erb*α reduces oxidative capacity, which causes exercise intolerance (Woldt et al., 2013). RORα is highly expressed in skeletal muscles (Becker-Andre et al., 1993). A deficiency in *Ror*α inhibits the expression of genes involved in lipid homeostasis in skeletal muscle cells (Lau et al., 2004). In particular, RORα directly regulates the transcription of *Carnitine palmitoyltransferase-1* (*Cpt-1*) and *Caveolin-3* (Cav3) (Lau et al., 2004). These data suggest that the molecular clock generates the circadian rhythm of metabolism in skeletal muscle and that the disruption of circadian rhythm occurs owing to metabolic dysfunction in skeletal muscle.

## The phase-shifted effects of exercise and feeding time on muscular circadian clocks

**Figure 1:**
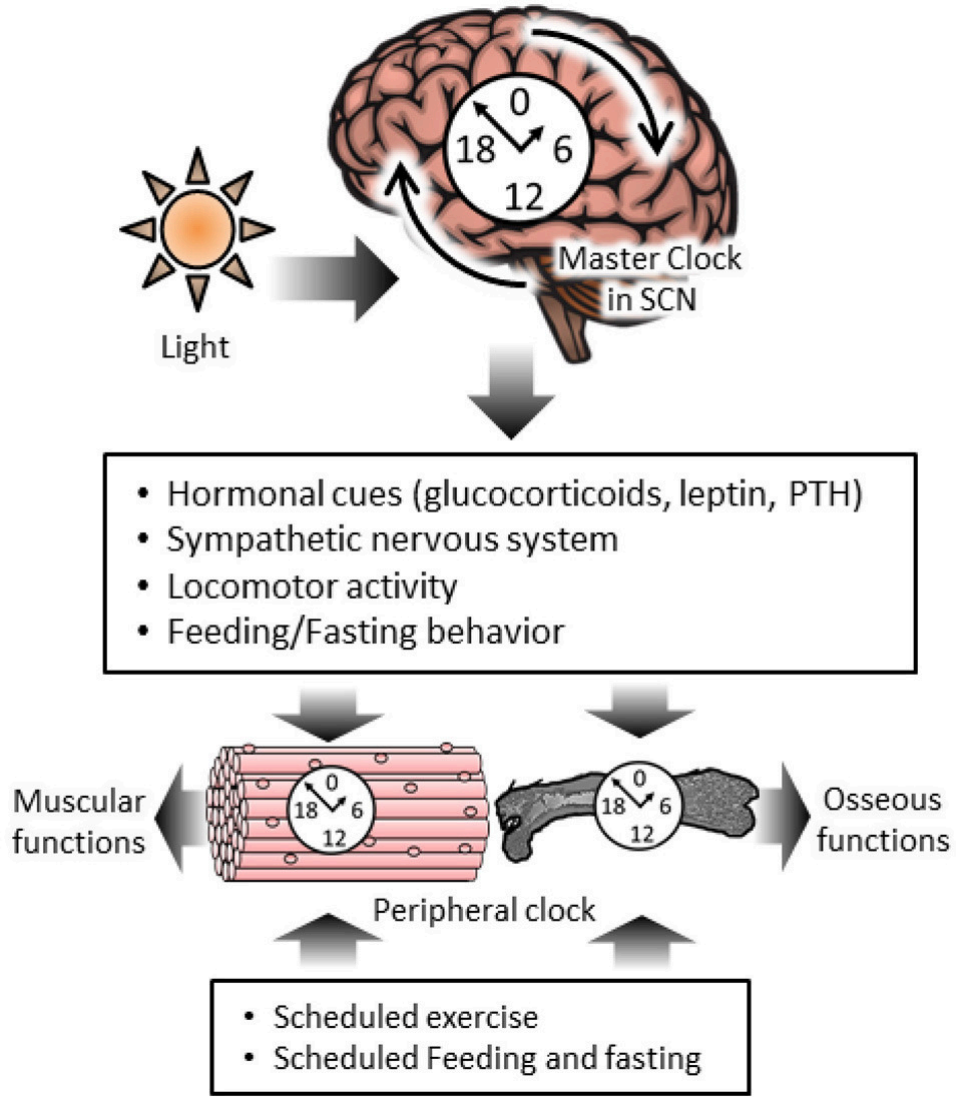
**The diurnal regulation of skeletal muscle and osseous functions by circadian clock systems and chrono-exercise and –nutrition**. The master clock in the suprachiasmatic nucleus (SCN) is reset by a photic cue and it regulates the peripheral circadian clock and homeostasis in skeletal muscle and bone via hormonal cues, the nervous system, locomotor activity and feeding behavior. Muscular clocks are regulated by scheduled exercise in an SCN-independent manner. Circadian variation of osseous markers are regulated by scheduled feeding.

The hierarchy of tissue-specific clocks exists in the mammalian circadian clock system. The clock in the SCN is termed the master clock while clocks in other brain areas, such as the cerebral cortex and hippocampus, and peripheral tissues, such as the liver and skeletal muscle, are termed brain clocks and peripheral clocks, respectively. Brain and peripheral clocks are under the control of the master clock. Classically, the master clock is entrained by a photic cue, such as the light-dark cycle, and it is thought that peripheral clocks are also regulated by the central clock in the SCN. On the other hand, peripheral clocks are controlled in a central-clock-independent manner under certain conditions, such as scheduled exercise and restricted feeding (Damiola et al., 2000; Stokkan et al., 2001). Exercise is one of the non-photic phase-shifting cues (Figure 1). Several studies have shown scheduled exercise during the daytime or subjective daytime advancing of the phase of circadian rhythms in rodents (Reebs and Mrosovsky, 1989; Marchant and Mistlberger, 1996). Wolff et al. have shown the phase advanced effects of scheduled exercise in *ex-vivo* experiments (Wolff and Esser, 2012). After scheduled voluntary or involuntary (treadmill running) exercise during the daytime for 4 weeks, the phase expression of *Per2::Luc* advances in skeletal muscle and lung but not in the SCN (Wolff and Esser, 2012). Scheduled exercise also accelerates re-entrainment in mouse skeletal muscle and lung but not liver or the SCN to a new light-dark cycle (Yamanaka et al., 2008). In addition, Yamanaka et al. have shown that exercise-induced re-entrainment depends on the timing of exercise and on the peripheral tissues (Yamanaka et al., 2016). In the phase advanced light/dark cycle condition, wheel running at the beginning of the active phase (onset) accelerates the re-entrainment of the skeletal muscle clock but not the SCN. On the other hand, in the phase delayed light/dark cycle condition, wheel running at the end of the active-phase (offset) interferes with the re-entrainment of the skeletal muscle clock. These reports have indicated the potential of exercise to induce a phase shifting effect in skeletal muscle with the use of *ex vivo* experiments. In recent years, we have reported that exercise advances the phase of circadian rhythm in peripheral clocks, such as liver and gastrocnemius muscle, in *in vivo* experiments (Sasaki et al., 2016). In this report, the exercise-induced phase advance of the liver clock was stronger following forced exercise rather than voluntary exercise, although the difference between the two kinds of exercise to cause entrainment was not observed in the skeletal muscle clock. While the mechanism of exercise-induced phase shifting has not been fully elucidated, the role of some factors has been proposed to date. Adrenocortical hormone (Balsalobre et al., 2000; Hayasaka et al., 2007; Sujino et al., 2012; Tahara et al., 2015) and the sympathetic nervous system (Terazono et al., 2003; Tahara et al., 2015) play roles as entraining factors of peripheral clocks, and they are released and activated by exercise (Fediuc et al., 1985; Chennaoui et al., 2002; Stranahan et al., 2008; Zouhal et al., 2008; Hansen et al., 2012). In fact, we have shown that forced treadmill running increases serum corticosterone and tissue norepinephrine levels and their elevations play an important role in the forced exercise-induced phase shifting of peripheral clocks (Sasaki et al., 2016). The effects of inactivity on peripheral clocks have also been reported (Dyar et al., 2015; Nakao et al., 2015). Nakao et al. showed that the expression of *Bmal1, Per1, Per2, Rora, Nr1d1*, and *Dbp* were decreased, and the expression of *Clock* was increased, in inactive muscles by denervation of the sciatic nerve (Nakao et al., 2015). In denervated muscles, *Bmal1* and *Dbp* showed phase advance, compared with the contralateral muscle. The denervation-induced phase advance of muscular clocks has also reported by Dyar et al. (2015). Although the loss of muscle activity did not completely abolish the rhythms of muscular clocks, the rhythmic expressions of many cyclic genes were altered by denervation (Dyar et al., 2015; Nakao et al., 2015). These data suggest that physical activity affects muscular clocks. Further investigation is needed into the role of neuronal signals and physical activity in the direct and indirect regulation of muscular clocks; physical activity causes several physiological changes, such as to body temperature and hormonal status, which are known to affect the peripheral clocks (Tahara et al., 2017). The phase shifting effects of exercise have been observed in human studies (Van Reeth et al., 1994; Buxton et al., 1997, 2003; Miyazaki et al., 2001; Barger et al., 2004). For example, Yamanaka et al. reported that the sleep-wake cycle but not melatonin rhythm is accelerated by exercise under dim light conditions and a phase-advanced sleep schedule (Yamanaka et al., 2010). Zambon et al. reported that resistant exercise changed the gene expression of the circadian clock in the skeletal muscle of humans (Zambon et al., 2003). These reports indicate that exercise acts as a potential entrainer of skeletal muscle clocks in humans as well as rodents.

As mentioned before, the peripheral clock is regulated by restricted feeding (Damiola et al., 2000; Stokkan et al., 2001). To date, restricted feeding shifts and entrains the liver clock in an SCN-independent manner (Hara et al., 2001; Tahara and Shibata, 2014). It is thought that insulin is the hormone responsible for the feeding-induced phase advance of the peripheral clock (Kuriyama et al., 2004; Tahara et al., 2011; Dang et al., 2016). Skeletal muscle is one of the major insulin sensitive organs and it controls whole body blood glucose levels via insulin-stimulated glucose uptake (DeFronzo, 1988). This evidence gives the expectation that clocks in insulin-sensitive organs are regulated by restricted feeding, in other words restricted feeding regulates the peripheral clock not only in the liver but also in skeletal muscle. However, Guo et al. reported tissue-dependent regulation of the peripheral clock by hormonal cues in SCN-lesioned mice (Guo et al., 2005). When circulating factors in SCN-lesioned mice and intact mice are shared by parabiosis, the arrhythmic clock gene expression in the liver of SCN-lesioned mice is restored. On the other hand, the disturbance of the skeletal muscle clock is not restored by parabiosis, indicating that circulating factors, such as hormones, are not important for the regulation of the skeletal muscle clock. In fact, some reports have shown that the expression of core clock genes, such as *Bmal1* and *Per2*, is synchronized by day time feeding in the liver but not skeletal muscle (Reznick et al., 2013; Yasumoto et al., 2016). On the other hand, some rhythmic genes, such as *Pdk4* (*pyruvate dehydrogenase kinase 4*) and *Ucp3*, in skeletal muscles show arrhythmic or dampened expression patterns owing to day time feeding. These data suggest that restricted feeding does not have a major effect on the skeletal muscle clock. However, further evidence is needed because reports about the effect of restricted feeding on circadian rhythm in skeletal muscle have been limited.

## Circadian rhythm of bone functions

Bone is one of the organs to play a major role in the storage of calcium and phosphorus. Three kinds of cells exist in bone, namely osteocytes, osteoblasts, and osteoclasts, (Prideaux et al., 2016). More than 90% of cells in bone are osteocytes, and these cells play a role in the storage of bone matrix (Prideaux et al., 2016), whereas osteoblasts and osteoclasts regulate bone remodeling. Bone homeostasis is regulated by the balance between the production of bone matrix by osteoblasts, called bone formation, and the breakdown of bone matrix by osteoclasts, called bone resorption (Rodan and Martin, 2000). Not only the process of bone remodeling but also serum concentrations of some hormones regulating bone metabolism show diurnal variation (Dudek and Meng, 2014). For example, it has been reported that serum concentrations of calcitonin, calcium, osteocalcin, parathyroid hormone C-telopeptide, skeletal alkaline phosphatase, and tartate-resistant acid phosphatase show diurnal variation (Greenspan et al., 1997; Srivastava et al., 2001; Shao et al., 2003; Yang and Meng, 2016). In addition, Zvonic et al. reported on the circadian transcriptome in calvarial bone (Zvonic et al., 2007). In this report, more than 26% of genes expressed in calvarial bone show a rhythmic expression (Zvonic et al., 2007). Interestingly, among the rhythmic genes in calvarial bone 64% of genes do not exhibit rhythmic expression in liver, brown adipose tissue or white adipose tissue. In other words, a lot of rhythmic gene expression is controlled in a tissue-specific manner. For example, calcium channels, ADAMs (a disintegrin and metalloproteinases), FGFs (fibroblast growth factors), and Runxs (runt related transcription factors), which have critical roles in bone formation, bone remodeling, bone metabolism regulated cytokines and bone-specific transcriptional factors, show diurnal gene expression patterns (Zvonic et al., 2007). The transcriptomic results from calvarial bone suggest that the process of metabolism in several bones is regulated by circadian rhythm.

Indeed, it has been reported that some molecular clocks regulate and maintain bone homeostasis (Table 1). Fu et al. demonstrated that both *Per1* deficient and *Per2* PAS domain mutant mice have high bone mass (Fu et al., 2005). This phenotype is also exhibited by other clock gene modified mice, i.e., *Per1* and *2* double knockout mice, *Cry1* and *2* double knockout mice, *Bmal1* knockout mice, *Per2* mutant mice and *Cry2* knockout mice (Fu et al., 2005; Maronde et al., 2010). These reports have suggested that the molecular clock is a negative regulator of bone mass. The high bone mass induced by molecular clock-deficiency is controlled by osteoblast differentiation via leptin-Adrβ2 (adrenoceptor beta 2)-Creb1 cascades (Fu et al., 2005). Indeed, leptin levels are elevated in mice with both genotypes, *Per1*^−/−^ and *Per2*^−/−^ *mutant* mice, and the elevation of leptin levels may be involved in osteoblast differentiation via Adrβ2 activation. In fact, it has been reported that both leptin deficient mice and *Adrb2* deficient mice display a low bone mass phenotype (Ducy et al., 2000; Takeda et al., 2002). Therefore, clock deficiency-induced high bone mass is involved in leptin-dependent sympathetic activation via the Adrβ2. In contrast, Samsa et al. reported that deficiency of *Bmal1* in mice results in age-related bone loss (Samsa et al., 2016), which is in contrast to the results of a previous report (Fu et al., 2005). The different bone phenotypes in these reports may be due to the age differences of the mice. *Pers, Crys*, or *Bmal1* deficiency-induced high bone mass is observed during young and adolescent ages (Fu et al., 2005; Maronde et al., 2010). On the other hand, *Bmal1* deficiency-induced low bone mass is observed at geriatric age (Samsa et al., 2016). *Bmal1* knockout mice also show age-associated phenotypes such as body weight loss and sarcopenia (Kondratov et al., 2006). In addition, abnormal bone calcification and arthropathy in *Bmal1* knockout mice did not replicate in tamoxifen-inducible *Bmal1* knockout mice after development (Yang et al., 2016). While the reason for the opposing effects of *Bmal1* on bone mass have not been well elucidated, the age-dependent effects of *Bmal1* on bone mass may be one explanation.

The effects of circadian clock genes on bone resorption have been examined using osteoclast-specific *Bmal1* knockout mice (Xu et al., 2016). The osteoclast-specific *Bmal1* knockout mice have a high bone mass. The upregulation of *Nfatc1* via the direct regulation of the heterodimer of BMAL1 and CLOCK induces osteoclast differentiation and then reduces bone mass (Xu et al., 2016). This report suggests that the osteoclast clock genes reduce bone mass via the activation of osteoclast differentiation.

Interestingly, in recent years it has been reported that skeletal muscle clocks are linked to the maintenance of bone homeostasis. Schroder et al. reported that muscle specific-*Bmal1* knockout mice show thickening of the distal tibia (Schroder et al., 2015). Muscle and bone may communicate with each other via endocrine factors and mechanical loading (Karsenty and Olson, 2016). For example, myostatin, one of the hormones secreted from skeletal muscle, regulates osteoclast differentiation (Dankbar et al., 2015). While the mechanism of interaction of these tissues has not been fully elucidated yet, the skeletal muscle clock is closely linked to the maintenance of bone homeostasis.

## Regulation of the osseous circadian rhythm by internal and external cues

The circadian rhythms of bone functions are controlled by internal or external cues to maintain a balance between bone formation and bone resorption. The master clock in the SCN controls the peripheral circadian clocks via output signals such as hormonal and sympathetic nervous system signaling. Leptin is known to be one of the entraining factors of the circadian clock in osteoblasts (Fu et al., 2005) (Figure 1). Leptin activates the sympathetic nervous system via the Adrβ2, of which Creb1 is a downstream factor (Fu et al., 2005). These reports indicate that the osteoblast clock is regulated by sympathetic nervous system signals. In addition, in human osteoblasts, treatment with a β-adrenergic receptor agonist, isoprenaline, or synthetic glucocorticoids, dexamethasone, induces the circadian expression of clock genes (Komoto et al., 2012), suggesting that both sympathetic nervous system and glucocorticoid signals are involved in the circadian rhythm in osteoblasts and act as mediators from the SCN to osteoblasts. On the other hand, in osteoclasts, glucocorticoid signals rather than sympathetic signals have the ability to regulate rhythmic gene expression. Fujihara et al. demonstrated that the rhythmic expression of clock genes is changed by stimulation with dexamethasone but not isoprenaline (Fujihara et al., 2014). Osteoclast-specific genes, *Nfatc1* (*nuclear factor of activated T cells 1*) and *Ctsk* (*cathepsin K*), show rhythmic expressions (Fujihara et al., 2014). The rhythmic expression of *Nfatc1* participates in *Bmal1*-regulated bone resorption, as described above (Xu et al., 2016). These rhythmic expressions are dampened in cancellous bone from adrenalectomized mice, and glucocorticoid injection restores the rhythmic expression of these genes (Fujihara et al., 2014). This evidence indicates that glucocorticoid signaling acts as a mediator between the SCN and osteoclasts for the synchronization of circadian rhythms in osteoclasts.

Feeding and fasting regulate the diurnal variation of the bone resorption marker serum C-telopeptide fragments of collagen type 1 degradation (s-CTx) (Gertz et al., 1998) (Figure 1). The levels of s-CTx in humans are higher during early morning, from 05:00 to 08:00, and lower in the late afternoon, from 12:00 to 16:00. The levels of s-CTx show diurnal variation under normal feeding conditions, such as the consumption of breakfast, lunch and dinner, while fasting dampens the diurnal variation of s-CTx (Bjarnason et al., 2002; Qvist et al., 2002). In addition, the feeding-induced generation of this diurnal variation is also observed in the intake of glucose, protein, and fat (Bjarnason et al., 2002). The diurnal variation of levels of s-CTx under normal feeding conditions only occurs during breakfast but not lunch or dinner (Bjarnason et al., 2002). A reason for this may be that fasting plays a role in the feeding-induced generation of the s-CTx rhythm, since the fasting period from breakfast to lunch and from lunch to dinner is shorter than that from dinner to breakfast. These reports suggest that the feeding and fasting rhythm is an important factor in regulating and maintaining the circadian rhythm of bone resorption, although the mechanism for the preventative effects of food intake on bone resorption has not been well investigated.

Mechanical loading, such as exercise, protects against age-related bone loss, whereas unloading, such as bed rest, induces bone loss (Qi et al., 2016). Some reports have shown the effects of unloading on circadian rhythms of bone resorption markers (Halloran et al., 1988; Pedersen et al., 1995; Kim et al., 2000). Pedersen et al. showed that 5 days of bed rest in healthy women did not change the circadian rhythm of s-CTx or other serum bone resorption markers, such as alkaline phosphatase and osteocalcin (Pedersen et al., 1995). No effects of unloading on diurnal variation of bone resorption markers have been shown by other reports (Halloran et al., 1988; Kim et al., 2000). These reports suggest that a common physical activity rhythm, which also includes low intensity exercise, does not have the potential to regulate the circadian rhythm of bone resorption. However, moderate or high intensity scheduled exercise may affect the circadian variation of bone metabolism, since mechanical loading influences bone resorption and bone formation, as well as circulating factors such as glucocorticoids and parathyroid hormone (Fragala et al., 2011; Gardinier et al., 2015; Qi et al., 2016; Sasaki et al., 2016).

## Perspectives

Based on recent findings, circadian rhythms in skeletal muscle and bone maintain their homeostasis. The disruption of muscle clocks occurs owing to dysregulation of whole body glucose metabolism (Harfmann et al., 2016) and bone clocks could be negative regulators of bone mass through the inhibition of bone formation and the activation of bone resorption (Fu et al., 2005; Xu et al., 2016). These finding suggest that disturbances of circadian rhythms by social or environmental factors, such as shift work, may result in dysfunctions of skeletal muscle and bone. In epidemiological studies, the prevalence of metabolic syndrome, osteoporosis and bone fractures is increased in shift workers (Feskanich et al., 2009; Pietroiusti et al., 2010; Quevedo and Zuniga, 2010). In addition, it has been reported that long term constant light exposure reduces muscle strength and bone mass in mice (Lucassen et al., 2016). These findings indicate that the regulation of circadian rhythms in skeletal muscle and bone by external cues, such as feeding and exercise, are important for the maintenance of homeostasis in these tissues, since circadian rhythm in these tissues can be entrained or regulated by the feeding/fasting rhythm and the physical activity rhythm, including scheduled exercise. We report that disturbances of circadian rhythms in peripheral clocks under constant light conditions are partially improved by scheduled feeding and scheduled exercise, although the circadian clocks in skeletal muscle and bone have not been measured (Hamaguchi et al., 2015). In addition, the reductions in both mitochondrial content and exercise tolerance in the skeletal muscle of *Clock* mutant mice are restored by endurance exercise training (Pastore and Hood, 2013), suggesting that exercise can prevent circadian disturbance-induced muscular dysfunctions. In addition, some reports have shown the importance of the circadian timing of exercise for the prevention of diseases. Schroeder et al. have reported that rhythmic deficits observed in vasointestinal polypeptide-deficient mice are improved by wheel running exercise (Schroeder et al., 2012). Interestingly, greater preventative effects are observed when wheel running occurs at the end of the active phase but not at the beginning of the active phase (Schroeder et al., 2012). We have also reported that wheel running exercise at the end of the active phase has more preventative effects on high fat diet-induced obesity than that occurring at the beginning of the active phase (Sasaki et al., 2014). In addition, exercise in the morning but not in the afternoon or evening increases fat oxidation over 24 h in healthy humans (Iwayama et al., 2015). These reports suggest the importance of scheduled feeding or exercise for skeletal muscle and bone health. However, the importance of the circadian timing of exercise and nutritional intake for muscular and osseous health has not been well elucidated. Further advanced evidence is required and it is expected to lead to a better understanding of the mutual interaction between the circadian clock and muscle/bone.

## Author contributions

SA and SS were involved in conceptualizing and writing the manuscript.

## Funding

This work was supported by the council for Science, Technology, and Innovation, SIP, “Technologies for creating next-generation agriculture, forestry, and fisheries” (funding agency: Bio-oriented Technology Research Advancement Institution, NARO) (SS).

### Conflict of interest statement

The authors declare that the research was conducted in the absence of any commercial or financial relationships that could be construed as a potential conflict of interest.

## References

[B1] Andrews J. L. Zhang X. McCarthy J. J. McDearmon E. L. Hornberger T. A. Russell B.. (2010). CLOCK and BMAL1 regulate MyoD and are necessary for maintenance of skeletal muscle phenotype and function. Proc. Natl. Acad. Sci. U.S.A. 107, 19090–19095. 10.1073/pnas.1014523107 20956306 PMC2973897

[B2] Bae K. Lee K. Seo Y. Lee H. Kim D. Choi I. (2006). Differential effects of two period genes on the physiology and proteomic profiles of mouse anterior tibialis muscles. Mol. Cells 22, 275–284. 17202855

[B3] Balsalobre A. Brown S. A. Marcacci L. Tronche F. Kellendonk C. Reichardt H. M.. (2000). Resetting of circadian time in peripheral tissues by glucocorticoid signaling. Science 289, 2344–2347. 10.1126/science.289.5488.2344 11009419

[B4] Barger L. K. Wright K. P. Jr. Hughes R. J. Czeisler C. A. (2004). Daily exercise facilitates phase delays of circadian melatonin rhythm in very dim light. Am. J. Physiol. Regul. Integr. Comp. Physiol. 286, R1077–R1084. 10.1152/ajpregu.00397.2003 15031136

[B5] Becker-Andre M. Andre E. DeLamarter J. F. (1993). Identification of nuclear receptor mRNAs by RT-PCR amplification of conserved zinc-finger motif sequences. Biochem. Biophys. Res. Commun. 194, 1371–1379. 7916608 10.1006/bbrc.1993.1976

[B6] Bjarnason N. H. Henriksen E. E. Alexandersen P. Christgau S. Henriksen D. B. Christiansen C. (2002). Mechanism of circadian variation in bone resorption. Bone 30, 307–313. 10.1016/S8756-3282(01)00662-7 11792602

[B7] Busino L. Bassermann F. Maiolica A. Lee C. Nolan P. M. Godinho S. I.. (2007). SCFFbxl3 controls the oscillation of the circadian clock by directing the degradation of cryptochrome proteins. Science 316, 900–904. 10.1126/science.1141194 17463251

[B8] Buxton O. M. Frank S. A. L'Hermite-Baleriaux M. Leproult R. Turek F. W. Van Cauter E. (1997). Roles of intensity and duration of nocturnal exercise in causing phase delays of human circadian rhythms. Am. J. Physiol. 273, E536–E542. 9316443 10.1152/ajpendo.1997.273.3.E536

[B9] Buxton O. M. Lee C. W. L'Hermite-Baleriaux M. Turek F. W. Van Cauter E. (2003). Exercise elicits phase shifts and acute alterations of melatonin that vary with circadian phase. Am. J. Physiol. Regul. Integr. Comp. Physiol. 284, R714–R724. 10.1152/ajpregu.00355.2002 12571075

[B10] Charge S. B. Brack A. S. Bayol S. A. Hughes S. M. (2008). MyoD- and nerve-dependent maintenance of MyoD expression in mature muscle fibres acts through the DRR/PRR element. BMC Dev. Biol. 8:5. 10.1186/1471-213X-8-5 18215268 PMC2259323

[B11] Chatterjee S. Ma K. (2016). Circadian clock regulation of skeletal muscle growth and repair. F1000Research 5:1549. 10.12688/f1000research.9076.1 27540471 PMC4965692

[B12] Chatterjee S. Nam D. Guo B. Kim J. M. Winnier G. E. Lee J.. (2013). Brain and muscle Arnt-like 1 is a key regulator of myogenesis. J. Cell Sci. 126, 2213–2224. 10.1242/jcs.120519 23525013 PMC3672937

[B13] Chatterjee S. Yin H. Nam D. Li Y. Ma K. (2015). Brain and muscle Arnt-like 1 promotes skeletal muscle regeneration through satellite cell expansion. Exp. Cell Res. 331, 200–210. 10.1016/j.yexcr.2014.08.041 25218946

[B14] Chennaoui M. Gomez Merino D. Lesage J. Drogou C. Guezennec C. Y. (2002). Effects of moderate and intensive training on the hypothalamo-pituitary-adrenal axis in rats. Acta Physiol. Scand. 175, 113–21. 10.1046/j.1365-201x.2002.00971.x 12028131

[B15] Damiola F. Le Minh N. Preitner N. Kornmann B. Fleury-Olela F. Schibler U. (2000). Restricted feeding uncouples circadian oscillators in peripheral tissues from the central pacemaker in the suprachiasmatic nucleus. Genes Dev. 14, 2950–2961. 10.1101/gad.183500 11114885 PMC317100

[B16] Dang F. Sun X. Ma X. Wu R. Zhang D. Chen Y.. (2016). Insulin post-transcriptionally modulates Bmal1 protein to affect the hepatic circadian clock. Nat. Commun. 7:12696. 10.1038/ncomms12696 27576939 PMC5013695

[B17] Dankbar B. Fennen M. Brunert D. Hayer S. Frank S. Wehmeyer C.. (2015). Myostatin is a direct regulator of osteoclast differentiation and its inhibition reduces inflammatory joint destruction in mice. Nat. Med. 21, 1085–1090. 10.1038/nm.3917 26236992

[B18] DeFronzo R. A. (1988). Lilly lecture 1987. The triumvirate:β-cell, muscle, liver. A collusion responsible for NIDDM. Diabetes 37, 667–687. 3289989 10.2337/diab.37.6.667

[B19] Dolatshad H. Cary A. J. Davis F. C. (2010). Differential expression of the circadian clock in maternal and embryonic tissues of mice. PLoS ONE 5:e9855. 10.1371/journal.pone.0009855 20352049 PMC2844431

[B20] Ducy P. Amling M. Takeda S. Priemel M. Schilling A. F. Beil F. T.. (2000). Leptin inhibits bone formation through a hypothalamic relay: a central control of bone mass. Cell 100, 197–207. 10.1016/s0092-8674(00)81558-5 10660043

[B21] Dudek M. Meng Q. J. (2014). Running on time: the role of circadian clocks in the musculoskeletal system. Biochem. J. 463, 1–8. 10.1042/BJ20140700 25195734 PMC4157581

[B22] Dyar K. A. Ciciliot S. Tagliazucchi G. M. Pallafacchina G. Tothova J. Argentini C.. (2015). The calcineurin-NFAT pathway controls activity-dependent circadian gene expression in slow skeletal muscle. Mol. Metab. 4, 823–833. 10.1016/j.molmet.2015.09.004 26629406 PMC4632177

[B23] Dyar K. A. Ciciliot S. Wright L. E. Bienso R. S. Tagliazucchi G. M. Patel V. R. (2014). Muscle insulin sensitivity and glucose metabolism are controlled by the intrinsic muscle clock. Mol. Metab. 3, 29–41. 10.1016/j.molmet.2013.10.005 24567902 PMC3929910

[B24] Fediuc S. Campbell J. E. Riddell M. C. (1985). Effect of voluntary wheel running on circadian corticosterone release and on HPA axis responsiveness to restraint stress in Sprague-Dawley rats. J. Appl. Physiol. 100, 1867–1875. 16439512 10.1152/japplphysiol.01416.2005

[B25] Feskanich D. Hankinson S. E. Schernhammer E. S. (2009). Nightshift work and fracture risk: the Nurses' Health Study. Osteoporos. Int. 20, 537–542. 10.1007/s00198-008-0729-5 18766292 PMC2651998

[B26] Fragala M. S. Kraemer W. J. Denegar C. R. Maresh C. M. Mastro A. M. Volek J. S. (2011). Neuroendocrine-immune interactions and responses to exercise. Sports Med. 41, 621–639. 10.2165/11590430-000000000-00000 21780849

[B27] Fu L. Patel M. S. Bradley A. Wagner E. F. Karsenty G. (2005). The molecular clock mediates leptin-regulated bone formation. Cell 122, 803–815. 10.1016/j.cell.2005.06.028 16143109

[B28] Fujihara Y. Kondo H. Noguchi T. Togari A. (2014). Glucocorticoids mediate circadian timing in peripheral osteoclasts resulting in the circadian expression rhythm of osteoclast-related genes. Bone 61, 1–9. 10.1016/j.bone.2013.12.026 24389417

[B29] Gardinier J. D. Mohamed F. Kohn D. H. (2015). PTH signaling during exercise contributes to bone adaptation. J. Bone Miner. Res. 30, 1053–1063. 10.1002/jbmr.2432 25529455 PMC4734644

[B30] Gekakis N. Staknis D. Nguyen H. B. Davis F. C. Wilsbacher L. D. King D. P.. (1998). Role of the CLOCK protein in the mammalian circadian mechanism. Science 280, 1564–1569. 10.1126/science.280.5369.1564 9616112

[B31] Gertz B. J. Clemens J. D. Holland S. D. Yuan W. Greenspan S. (1998). Application of a new serum assay for type I collagen cross-linked N-telopeptides: assessment of diurnal changes in bone turnover with and without alendronate treatment. Calcif. Tissue Int. 63, 102–106. 10.1007/s002239900497 9685512

[B32] Greenspan S. L. Dresner-Pollak R. Parker R. A. London D. Ferguson L. (1997). Diurnal variation of bone mineral turnover in elderly men and women. Calcif. Tissue Int. 60, 419–423. 10.1007/s002239900256 9115158

[B33] Guo H. Brewer J. M. Champhekar A. Harris R. B. Bittman E. L. (2005). Differential control of peripheral circadian rhythms by suprachiasmatic-dependent neural signals. Proc. Natl. Acad. Sci. U.S.A. 102, 3111–3116. 10.1073/pnas.0409734102 15710878 PMC548796

[B34] Halloran B. P. Bikle D. D. Cone C. M. Morey-Holton E. (1988). Glucocorticoids and inhibition of bone formation induced by skeletal unloading. Am. J. Physiol. 255, E875–E879. 3202163 10.1152/ajpendo.1988.255.6.E875

[B35] Hamaguchi Y. Tahara Y. Hitosugi M. Shibata S. (2015). Impairment of circadian rhythms in peripheral clocks by constant light is partially reversed by scheduled feeding or exercise. J. Biol. Rhythms 30, 533–542. 10.1177/0748730415609727 26467286

[B36] Hansen D. Meeusen R. Mullens A. Dendale P. (2012). Effect of acute endurance and resistance exercise on endocrine hormones directly related to lipolysis and skeletal muscle protein synthesis in adult individuals with obesity. Sports Med. 42, 415–431. 10.2165/11599590-000000000-00000 22455310

[B37] Hara R. Wan K. Wakamatsu H. Aida R. Moriya T. Akiyama M.. (2001). Restricted feeding entrains liver clock without participation of the suprachiasmatic nucleus. Genes Cells 6, 269–278. 10.1046/j.1365-2443.2001.00419.x 11260270

[B38] Harfmann B. D. Schroder E. A. Kachman M. T. Hodge B. A. Zhang X. Esser K. A. (2016). Muscle-specific loss of Bmal1 leads to disrupted tissue glucose metabolism and systemic glucose homeostasis. Skelet. Muscle 6:12. 10.1186/s13395-016-0082-x 27486508 PMC4969979

[B39] Hayasaka N. Yaita T. Kuwaki T. Honma S. Honma K. Kudo T.. (2007). Optimization of dosing schedule of daily inhalant dexamethasone to minimize phase shifting of clock gene expression rhythm in the lungs of the asthma mouse model. Endocrinology 148, 3316–3326. 10.1210/en.2007-0010 17412811

[B40] Hodge B. A. Wen Y. Riley L. A. Zhang X. England J. H. Harfmann B. D.. (2015). The endogenous molecular clock orchestrates the temporal separation of substrate metabolism in skeletal muscle. Skelet. Muscle 5:17. 10.1186/s13395-015-0039-5 26000164 PMC4440511

[B41] Hughes S. M. Koishi K. Rudnicki M. Maggs A. M. (1997). MyoD protein is differentially accumulated in fast and slow skeletal muscle fibres and required for normal fibre type balance in rodents. Mech. Dev. 61, 151–163. 10.1016/s0925-4773(96)00631-4 9076685

[B42] Iwayama K. Kurihara R. Nabekura Y. Kawabuchi R. Park I. Kobayashi M.. (2015). Exercise increases 24-h fat oxidation only when it is performed before breakfast. EBioMedicine 2, 2003–2009. 10.1016/j.ebiom.2015.10.029 26844280 PMC4703705

[B43] Karsenty G. Olson E. N. (2016). Bone and muscle endocrine functions: unexpected paradigms of inter-organ communication. Cell 164, 1248–1256. 10.1016/j.cell.2016.02.043 26967290 PMC4797632

[B44] Kim C. S. Maekawa Y. Fujita M. Sato N. Nishimuta M. Ishizaki Y. (2000). Immobilization on the day 14th does not disrupts the basic diurnal rhythm of bone resorption. J. Gravit. physiol. 7, P125–P126. 12697501

[B45] King D. P. Zhao Y. Sangoram A. M. Wilsbacher L. D. Tanaka M. Antoch M. P.. (1997). Positional cloning of the mouse circadian clock gene. Cell 89, 641–653. 10.1016/s0092-8674(00)80245-7 9160755 PMC3815553

[B46] Komoto S. Kondo H. Fukuta O. Togari A. (2012). Comparison of beta-adrenergic and glucocorticoid signaling on clock gene and osteoblast-related gene expressions in human osteoblast. Chronobiol. Int. 29, 66–74. 10.3109/07420528.2011.636496 22217103

[B47] Kondratov R. V. Kondratova A. A. Gorbacheva V. Y. Vykhovanets O. V. Antoch M. P. (2006). Early aging and age-related pathologies in mice deficient in BMAL1, the core componentof the circadian clock. Genes Dev. 20, 1868–1873. 10.1101/gad.1432206 16847346 PMC1522083

[B48] Kuriyama K. Sasahara K. Kudo T. Shibata S. (2004). Daily injection of insulin attenuated impairment of liver circadian clock oscillation in the streptozotocin-treated diabetic mouse. FEBS Lett. 572, 206–210. 10.1016/j.febslet.2004.07.036 15304349

[B49] Lau P. Nixon S. J. Parton R. G. Muscat G. E. (2004). RORα regulates the expression of genes involved in lipid homeostasis in skeletal muscle cells: caveolin-3 and CPT-1 are direct targets of ROR. J. Biol. Chem. 279, 36828–36840. 10.1074/jbc.M404927200 15199055

[B50] Lee S. J. Huynh T. V. Lee Y. S. Sebald S. M. Wilcox-Adelman S. A. Iwamori N.. (2012). Role of satellite cells versus myofibers in muscle hypertrophy induced by inhibition of the myostatin/activin signaling pathway. Proc. Natl. Acad. Sci. U.S.A. 109, E2353–E2360. 10.1073/pnas.1206410109 22869749 PMC3435227

[B51] Legerlotz K. Smith H. K. (2008). Role of MyoD in denervated, disused, and exercised muscle. Muscle Nerve 38, 1087–1100. 10.1002/mus.21087 18642380

[B52] Lipton J. O. Yuan E. D. Boyle L. M. Ebrahimi-Fakhari D. Kwiatkowski E. Nathan A.. (2015). The circadian protein BMAL1 regulates translation in response to S6K1-mediated phosphorylation. Cell 161, 1138–1151. 10.1016/j.cell.2015.04.002 25981667 PMC4447213

[B53] Liu J. Zhou B. Yan M. Huang R. Wang Y. He Z.. (2016). CLOCK and BMAL1 regulate muscle insulin sensitivity via SIRT1 in male mice. Endocrinology 157, 2259–2269. 10.1210/en.2015-2027 27035655

[B54] Lowrey P. L. Shimomura K. Antoch M. P. Yamazaki S. Zemenides P. D. Ralph M. R.. (2000). Positional syntenic cloning and functional characterization of the mammalian circadian mutation tau. Science 288, 483–492. 10.1126/science.288.5465.483 10775102 PMC3869379

[B55] Lucassen E. A. Coomans C. P. van Putten M. de Kreij S. R. van Genugten J. H. Sutorius R. P.. (2016). Environmental 24-hr cycles are essential for health. Curr. Biol. 26, 1843–1853. 10.1016/j.cub.2016.05.038 27426518

[B56] Marchant E. G. Mistlberger R. E. (1996). Entrainment and phase shifting of circadian rhythms in mice by forced treadmill running. Physiol. Behav. 60, 657–663. 10.1016/S0031-9384(96)80045-X 8840932

[B57] Maronde E. Schilling A. F. Seitz S. Schinke T. Schmutz I. van der Horst G.. (2010). The clock genes Period 2 and Cryptochrome 2 differentially balance bone formation. PLoS ONE 5:e11527. 10.1371/journal.pone.0011527 20634945 PMC2902506

[B58] Mayeuf-Louchart A. Staels B. Duez H. (2015). Skeletal muscle functions around the clock. Diabetes Obes. Metab. 17(Suppl. 1), 39–46. 10.1111/dom.12517 26332967

[B59] McCarthy J. J. Andrews J. L. McDearmon E. L. Campbell K. S. Barber B. K. Miller B. H.. (2007). Identification of the circadian transcriptome in adult mouse skeletal muscle. Physiol. Genomics 31, 86–95. 10.1152/physiolgenomics.00066.2007 17550994 PMC6080860

[B60] McDearmon E. L. Patel K. N. Ko C. H. Walisser J. A. Schook A. C. Chong J. L.. (2006). Dissecting the functions of the mammalian clock protein BMAL1 by tissue-specific rescue in mice. Science 314, 1304–1308. 10.1126/science.1132430 17124323 PMC3756687

[B61] Miller B. H. McDearmon E. L. Panda S. Hayes K. R. Zhang J. Andrews J. L.. (2007). Circadian and CLOCK-controlled regulation of the mouse transcriptome and cell proliferation. Proc. Natl. Acad. Sci. U.S.A. 104, 3342–3347. 10.1073/pnas.0611724104 17360649 PMC1802006

[B62] Miyazaki T. Hashimoto S. Masubuchi S. Honma S. Honma K. I. (2001). Phase-advance shifts of human circadian pacemaker are accelerated by daytime physical exercise. Am. J. Physiol. Regul. Integr. Comp. Physiol. 281, R197–R205. 11404294 10.1152/ajpregu.2001.281.1.R197

[B63] Nakao R. Yamamoto S. Horikawa K. Yasumoto Y. Nikawa T. Mukai C.. (2015). Atypical expression of circadian clock genes in denervated mouse skeletal muscle. Chronobiol. Int. 32, 486–496. 10.3109/07420528.2014.1003350 25798696

[B64] Pastore S. Hood D. A. (2013). Endurance training ameliorates the metabolic and performance characteristics of circadian Clock mutant mice. J. Appl. Physiol. 14, 1076–1084. 10.1152/japplphysiol.01505.2012 23429867

[B65] Pedersen B. J. Schlemmer A. Hassager C. Christiansen C. (1995). Changes in the carboxyl-terminal propeptide of type I procollagen and other markers of bone formation upon five days of bed rest. Bone 17, 91–95. 10.1016/8756-3282(95)00149-8 7577164

[B66] Pietroiusti A. Neri A. Somma G. Coppeta L. Iavicoli I. Bergamaschi A.. (2010). Incidence of metabolic syndrome among night-shift healthcare workers. Occup. Environ. Med. 67, 54–57. 10.1136/oem.2009.046797 19737731

[B67] Pircher P. Chomez P. Yu F. Vennstrom B. Larsson L. (2005). Aberrant expression of myosin isoforms in skeletal muscles from mice lacking the rev-erbAα orphan receptor gene. Am. J. Physiol. Regul. Integr. Comp. Physiol. 288, R482–R490. 10.1152/ajpregu.00690.2003 15374821

[B68] Powers S. K. Kavazis A. N. DeRuisseau K. C. (2005). Mechanisms of disuse muscle atrophy: role of oxidative stress. Am. J. Physiol. Regul. Integr. Comp. Physiol. 288, R337–R344. 10.1152/ajpregu.00469.2004 15637170

[B69] Preitner N. Damiola F. Lopez-Molina L. Zakany J. Duboule D. Albrecht U.. (2002). The orphan nuclear receptor REV-ERBα controls circadian transcription within the positive limb of the mammalian circadian oscillator. Cell 110, 251–260. 10.1016/S0092-8674(02)00825-5 12150932

[B70] Prideaux M. Findlay D. M. Atkins G. J. (2016). Osteocytes: the master cells in bone remodelling. Curr. Opin. Pharmacol. 28, 24–30. 10.1016/j.coph.2016.02.003 26927500

[B71] Qi Z. Liu W. Lu J. (2016). The mechanisms underlying the beneficial effects of exercise on bone remodeling: roles of bone-derived cytokines and microRNAs. Prog. Biophys. Mol. Biol. 122, 131–139. 10.1016/j.pbiomolbio.2016.05.010 27179638

[B72] Quevedo I. Zuniga A. M. (2010). Low bone mineral density in rotating-shift workers. J. Clin. Densitom. 13, 467–469. 10.1016/j.jocd.2010.07.004 21029978

[B73] Qvist P. Christgau S. Pedersen B. J. Schlemmer A. Christiansen C. (2002). Circadian variation in the serum concentration of C-terminal telopeptide of type I collagen (serum CTx): effects of gender, age, menopausal status, posture, daylight, serum cortisol, and fasting. Bone 31, 57–61. 10.1016/s8756-3282(02)00791-3 12110413

[B74] Reebs S. G. Mrosovsky N. (1989). Effects of induced wheel running on the circadian activity rhythms of Syrian hamsters: entrainment and phase response curve. J. Biol. Rhythms 4, 39–48. 10.1177/074873048900400103 2519579

[B75] Reznick J. Preston E. Wilks D. L. Beale S. M. Turner N. Cooney G. J. (2013). Altered feeding differentially regulates circadian rhythms and energy metabolism in liver and muscle of rats. Biochim. Biophys. Acta 1832, 228–238. 10.1016/j.bbadis.2012.08.010 22952003

[B76] Rodan G. A. Martin T. J. (2000). Therapeutic approaches to bone diseases. Science 289, 1508–1514. 10.1126/science.289.5484.1508 10968781

[B77] Samsa W. E. Vasanji A. Midura R. J. Kondratov R. V. (2016). Deficiency of circadian clock protein BMAL1 in mice results in a low bone mass phenotype. Bone 84, 194–203. 10.1016/j.bone.2016.01.006 26789548 PMC4755907

[B78] Sasaki H. Hattori Y. Ikeda Y. Kamagata M. Iwami S. Yasuda S.. (2016). Forced rather than voluntary exercise entrains peripheral clocks via a corticosterone/noradrenaline increase in PER2::LUC mice. Sci. Rep. 6:27607. 10.1038/srep27607 27271267 PMC4897787

[B79] Sasaki H. Ohtsu T. Ikeda Y. Tsubosaka M. Shibata S. (2014). Combination of meal and exercise timing with a high-fat diet influences energy expenditure and obesity in mice. Chronobiol. Int. 31, 959–975. 10.3109/07420528.2014.935785 25007387

[B80] Sato T. K. Panda S. Miraglia L. J. Reyes T. M. Rudic R. D. McNamara P.. (2004). A functional genomics strategy reveals Rora as a component of the mammalian circadian clock. Neuron 43, 527–537. 10.1016/j.neuron.2004.07.018 15312651

[B81] Schiaffino S. Blaauw B. Dyar K. A. (2016). The functional significance of the skeletal muscle clock: lessons from Bmal1 knockout models. Skelet. Muscle 6:33. 10.1186/s13395-016-0107-5 27752300 PMC5062818

[B82] Schiaffino S. Reggiani C. (1996). Molecular diversity of myofibrillar proteins: gene regulation and functional significance. Physiol. Rev. 76, 371–423. 8618961 10.1152/physrev.1996.76.2.371

[B83] Schibler U. Ripperger J. Brown S. A. (2003). Peripheral circadian oscillators in mammals: time and food. J. Biol. Rhythms 18, 250–260. 10.1177/0748730403018003007 12828282

[B84] Schroder E. A. Harfmann B. D. Zhang X. Srikuea R. England J. H. Hodge B. A.. (2015). Intrinsic muscle clock is necessary for musculoskeletal health. J. Physiol. 593, 5387–5404. 10.1113/jp271436 26486627 PMC4704520

[B85] Schroeder A. M. Truong D. Loh D. H. Jordan M. C. Roos K. P. Colwell C. S. (2012). Voluntary scheduled exercise alters diurnal rhythms of behaviour, physiology and gene expression in wild-type and vasoactive intestinal peptide-deficient mice. J. Physiol. 590, 6213–6226. 10.1113/jphysiol.2012.233676 22988135 PMC3530127

[B86] Shao P. Ohtsuka-Isoya M. Shinoda H. (2003). Circadian rhythms in serum bone markers and their relation to the effect of etidronate in rats. Chronobiol. Int. 20, 325–336. 10.1081/cbi-120019343 12723888

[B87] Shavlakadze T. Anwari T. Soffe Z. Cozens G. Mark P. J. Gondro C.. (2013). Impact of fasting on the rhythmic expression of myogenic and metabolic factors in skeletal muscle of adult mice. Am. J. Physiol., Cell Physiol. 305, C26–C35. 10.1152/ajpcell.00027.2013 23596176

[B88] Shibata S. (2004). Neural regulation of the hepatic circadian rhythm. Anat. Rec. A Discov. Mol. Cell. Evol. Biol. 280, 901–909. 10.1002/ar.a.20095 15382011

[B89] Srivastava A. K. Bhattacharyya S. Li X. Mohan S. Baylink D. J. (2001). Circadian and longitudinal variation of serum C-telopeptide, osteocalcin, and skeletal alkaline phosphatase in C3H/HeJ mice. Bone 29, 361–367. 10.1016/s8756-3282(01)00581-6 11595619

[B90] Stokkan K. A. Yamazaki S. Tei H. Sakaki Y. Menaker M. (2001). Entrainment of the circadian clock in the liver by feeding. Science 291, 490–493. 10.1126/science.291.5503.490 11161204

[B91] Stranahan A. M. Lee K. Mattson M. P. (2008). Central mechanisms of HPA axis regulation by voluntary exercise. Neuromolecular Med. 10, 118–127. 10.1007/s12017-008-8027-0 18273712 PMC3010733

[B92] Sujino M. Furukawa K. Koinuma S. Fujioka A. Nagano M. Iigo M.. (2012). Differential entrainment of peripheral clocks in the rat by glucocorticoid and feeding. Endocrinology 153, 2277–2286. 10.1210/en.2011-1794 22434077

[B93] Tahara Y. Aoyama S. Shibata S. (2017). The mammalian circadian clock and its entrainment by stress and exercise. J. Physiol. Sci. 67, 1–10. 10.1007/s12576-016-0450-7 27084533 PMC5138246

[B94] Tahara Y. Otsuka M. Fuse Y. Hirao A. Shibata S. (2011). Refeeding after fasting elicits insulin-dependent regulation of Per2 and Rev-erbalpha with shifts in the liver clock. J. Biol. Rhythms 26, 230–240. 10.1177/0748730411405958 21628550

[B95] Tahara Y. Shibata S. (2013). Chronobiology and nutrition. Neuroscience 253, 78–88. 10.1016/j.neuroscience.2013.08.049 24007937

[B96] Tahara Y. Shibata S. (2014). Chrono-biology, chrono-pharmacology, and chrono-nutrition. J. Pharmacol. Sci. 124, 320–335. 10.1254/jphs.13r06cr 24572815

[B97] Tahara Y. Shibata S. (2016). Circadian rhythms of liver physiology and disease: experimental and clinical evidence. Nat. Rev. Gastroenterol. Hepatol. 13, 217–226. 10.1038/nrgastro.2016.8 26907879

[B98] Tahara Y. Shiraishi T. Kikuchi Y. Haraguchi A. Kuriki D. Sasaki H.. (2015). Entrainment of the mouse circadian clock by sub-acute physical and psychological stress. Sci. Rep. 5:11417. 10.1038/srep11417 26073568 PMC4466793

[B99] Takeda S. Elefteriou F. Levasseur R. Liu X. Zhao L. Parker K. L.. (2002). Leptin regulates bone formation via the sympathetic nervous system. Cell 111, 305–317. 10.1016/s0092-8674(02)01049-8 12419242

[B100] Terazono H. Mutoh T. Yamaguchi S. Kobayashi M. Akiyama M. Udo R.. (2003). Adrenergic regulation of clock gene expression in mouse liver. Proc. Natl. Acad. Sci. U.S.A. 100, 6795–6800. 10.1073/pnas.0936797100 12754374 PMC164526

[B101] Van Reeth O. Sturis J. Byrne M. M. Blackman J. D. L'Hermite-Baleriaux M. Leproult R.. (1994). Nocturnal exercise phase delays circadian rhythms of melatonin and thyrotropin secretion in normal men. Am. J. Physiol. 266, E964–E974. 8023928 10.1152/ajpendo.1994.266.6.E964

[B102] Voytik S. L. Przyborski M. Badylak S. F. Konieczny S. F. (1993). Differential expression of muscle regulatory factor genes in normal and denervated adult rat hindlimb muscles. Dev. Dyn. 198, 214–224. 10.1002/aja.1001980307 8136525

[B103] Woldt E. Sebti Y. Solt L. A. Duhem C. Lancel S. Eeckhoute J.. (2013). Rev-erb- α modulates skeletal muscle oxidative capacity by regulating mitochondrial biogenesis and autophagy. Nat. Med. 19, 1039–1046. 10.1038/nm.3213 23852339 PMC3737409

[B104] Wolff G. Esser K. A. (2012). Scheduled exercise phase shifts the circadian clock in skeletal muscle. Med. Sci. Sports Exerc. 44, 1663–1670. 10.1249/mss.0b013e318255cf4c 22460470 PMC3414645

[B105] Xu C. Ochi H. Fukuda T. Sato S. Sunamura S. Takarada T.. (2016). Circadian clock regulates bone resorption in mice. J. Bone Miner. Res. 31, 1344–1355. 10.1002/jbmr.2803 26841172

[B106] Yamanaka Y. Hashimoto S. Tanahashi Y. Nishide S. Y. Honma S. Honma K. (2010). Physical exercise accelerates reentrainment of human sleep-wake cycle but not of plasma melatonin rhythm to 8-h phase-advanced sleep schedule. Am. J. Physiol. Regul. Integr. Comp. Physiol. 298, R681–R691. 10.1152/ajpregu.00345.2009 20042689

[B107] Yamanaka Y. Honma S. Honma K. (2008). Scheduled exposures to a novel environment with a running-wheel differentially accelerate re-entrainment of mice peripheral clocks to new light-dark cycles. Genes Cells 13, 497–507. 10.1111/j.1365-2443.2008.01183.x 18429821

[B108] Yamanaka Y. Honma S. Honma K. (2016). Mistimed wheel running interferes with re-entrainment of circadian Per1 rhythms in the mouse skeletal muscle and lung. Genes Cells 21, 264–274. 10.1111/gtc.12336 26818910

[B109] Yang G. Chen L. Grant G. R. Paschos G. Song W. L. Musiek E. S.. (2016). Timing of expression of the core clock gene Bmal1 influences its effects on aging and survival. Sci. Transl. Med. 8:324ra16. 10.1126/scitranslmed.aad3305 26843191 PMC4870001

[B110] Yang N. Meng Q. J. (2016). Circadian clocks in articular cartilage and bone: a compass in the sea of matrices. J. Biol. Rhythms 31, 15–27. 10.1177/0748730416662748 27558096

[B111] Yasumoto Y. Hashimoto C. Nakao R. Yamazaki H. Hiroyama H. Nemoto T.. (2016). Short-term feeding at the wrong time is sufficient to desynchronize peripheral clocks and induce obesity with hyperphagia, physical inactivity and metabolic disorders in mice. Metab. Clin. Exp. 65, 714–727. 10.1016/j.metabol.2016.02.003 27085778

[B112] Zambon A. C. McDearmon E. L. Salomonis N. Vranizan K. M. Johansen K. L. Adey D.. (2003). Time- and exercise-dependent gene regulation in human skeletal muscle. Genome Biol. 4:R61. 10.1186/gb-2003-4-10-r61 14519196 PMC328450

[B113] Zhang R. Lahens N. F. Ballance H. I. Hughes M. E. Hogenesch J. B. (2014). A circadian gene expression atlas in mammals: implications for biology and medicine. Proc. Natl. Acad. Sci. U.S.A. 111, 16219–16224. 10.1073/pnas.1408886111 25349387 PMC4234565

[B114] Zhang X. Patel S. P. McCarthy J. J. Rabchevsky A. G. Goldhamer D. J. Esser K. A. (2012). A non-canonical E-box within the MyoD core enhancer is necessary for circadian expression in skeletal muscle. Nucleic Acids Res. 40, 3419–3430. 10.1093/nar/gkr1297 22210883 PMC3333858

[B115] Zouhal H. Jacob C. Delamarche P. Gratas-Delamarche A. (2008). Catecholamines and the effects of exercise, training and gender. Sports Med. 38, 401–423. 10.2165/00007256-200838050-00004 18416594

[B116] Zvonic S. Ptitsyn A. A. Kilroy G. Wu X. Conrad S. A. Scott L. K.. (2007). Circadian oscillation of gene expression in murine calvarial bone. J. Bone Miner. Res. 22, 357–365. 10.1359/jbmr.061114 17144790

